# Case Report: COVID-19 unmasks factor H mutation-driven hemolytic uremic syndrome in a previously undiagnosed septuagenarian kidney transplant recipient

**DOI:** 10.3389/fmed.2026.1702864

**Published:** 2026-03-31

**Authors:** Michael Fink, Marion Pollheimer, Alexander Kirsch, Alexander R. Rosenkranz, Kathrin Eller, Max Schuller

**Affiliations:** 1 Division of Nephrology, Department of Internal Medicine, Medical University of Graz, Graz, Austria; 2 Institute of Pathology, Medical University of Graz, Graz, Austria

**Keywords:** atypical HUS, complement-mediated thrombotic microangiopathy, COVID-19, factor H (FH), kidney transplantation, ravulizumab, thrombotic microangiopathy (TMA)

## Abstract

Thrombotic microangiopathy (TMA) after kidney transplantation presents a significant diagnostic and therapeutic challenge. Complement-mediated thrombotic microangiopathy (CM-TMA), caused by dysregulation of the alternative complement pathway, is frequently associated with predisposing variants in complement-regulatory genes but typically requires additional triggers for clinical manifestation. Ravulizumab, a long-acting terminal complement inhibitor, has demonstrated efficacy in native kidney disease, but data in transplant recipients remain limited. We present a 75-year-old kidney transplant recipient in whom previously unrecognized CM-TMA was triggered by COVID-19 infection and successfully treated with ravulizumab. This case underscores the importance of considering CM-TMA in the differential diagnosis of posttransplant TMA and in patients with end-stage kidney disease of uncertain origin, demonstrating that age alone should not preclude diagnostic consideration, and supports ravulizumab as an effective therapeutic option in this setting.

## Background

Thrombotic microangiopathy (TMA) is a rare condition characterized by microangiopathic hemolytic anemia, thrombocytopenia, and end-organ damage (1). Complement-mediated thrombotic microangiopathy (CM-TMA, formerly known as atypical hemolytic uremic syndrome) is a subtype of TMA caused by dysregulation of the alternative complement pathway.

Genetic variants in complement regulatory proteins are identified in up to 60% of CM-TMA cases. However, these variants are considered predisposing rather than directly causative, and a second hit, such as viral infection, is often required for disease manifestation (2). Among these, mutations in factor H account for approximately 25–30% of cases and may lead to either a quantitative or qualitative deficiency (3). Factor H plays a central role in complement regulation as a key negative regulator of alternative pathway activity and protects endothelial surfaces from complement-mediated injury (4).

The kidneys may be particularly vulnerable to complement-mediated injury because of their fenestrated endothelium and relatively low baseline levels of regulatory proteins (5). TMA can affect both native and transplanted kidneys, and the post-transplant setting appears particularly conducive to its development due to factors such as ischemic injury, viral infections, and immunosuppressive therapy (2). Given the condition’s high morbidity and mortality, early recognition and prompt treatment with complement inhibitors are essential (1).

Ravulizumab is a long-acting monoclonal antibody targeting complement component C5 and thereby preventing formation of the membrane attack complex. It binds the same epitope as eculizumab with similar affinity but has an extended half-life (6). Although ravulizumab has been approved for the treatment of CM-TMA, experience in the post-transplant setting remains limited, with data currently restricted to a small cohort of eight kidney transplant recipients (6).

## Case report

A 75-year-old patient underwent kidney transplantation from a deceased donor. Although not biopsy-proven, the suspected underlying renal pathology was hypertensive nephropathy due to long-standing arterial hypertension. Otherwise, the patient’s medical history was unremarkable. His family history revealed that his sister had also undergone kidney transplantation. Immunosuppression was initiated with basiliximab induction, followed by triple maintenance therapy comprising tacrolimus, mycophenolic acid, and prednisolone. After an uncomplicated immediate post-transplant period, the patient was discharged with a stable serum creatinine of approximately 2 mg/dL.

Three months after transplantation, a kidney biopsy was performed to rule out rejection after an increase in serum creatinine and showed acute tubular injury. Kidney function improved following optimized hemodynamic control.

**Figure 1:**
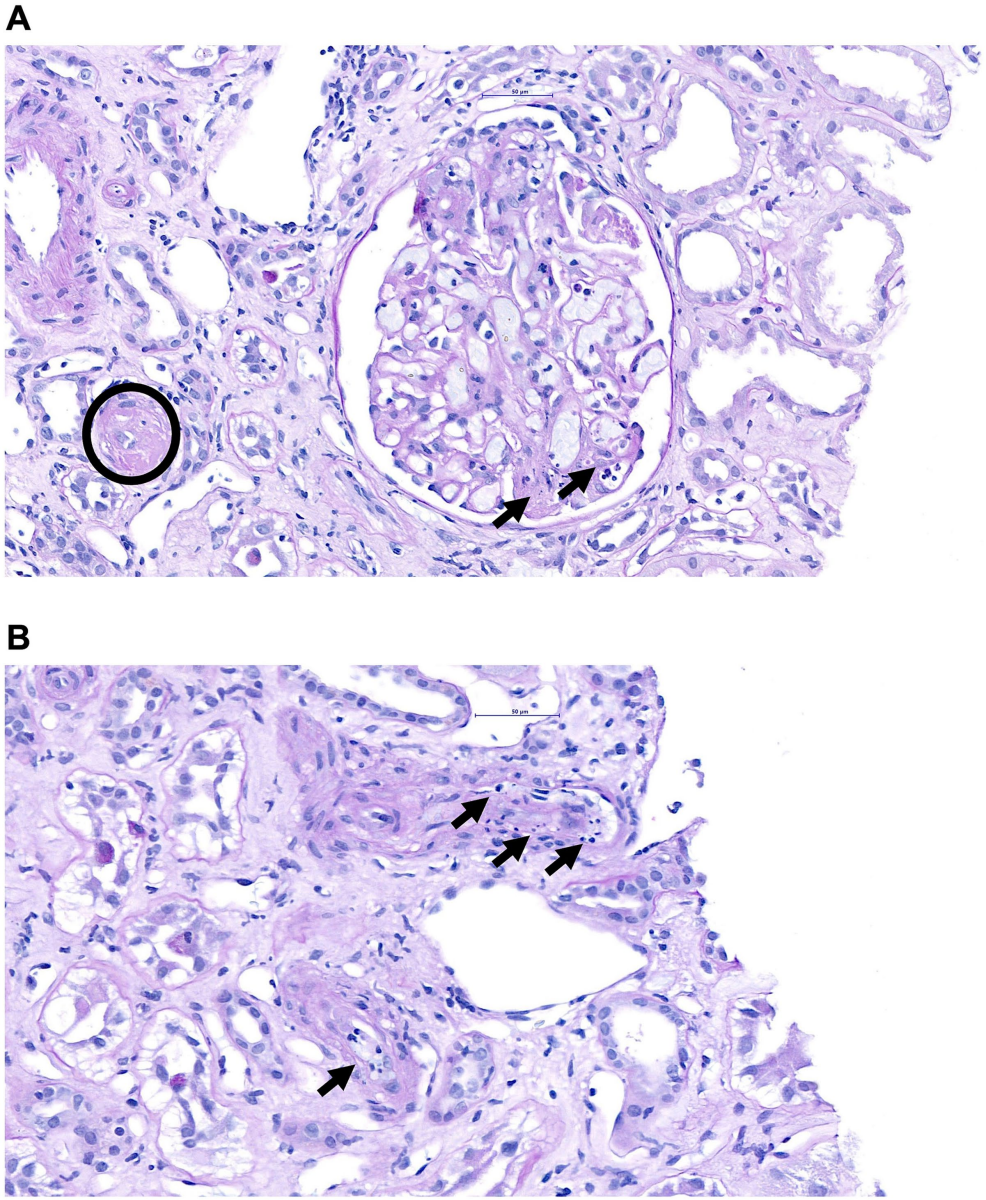
Periodic acid–Schiff (PAS)-stained kidney sections showing a glomerulus with segmental fibrinoid necrosis, mesangiolysis, and neutrophils/karyorrhexis (arrows). Note the arteriole with the fibrinoid necrosis (circle) **(A)**. PAS-stained section showing two small arteries with endothelial inflammation and swelling, as well as fibrinoid necrosis (arrows) **(B)**.

In October 2024, the patient presented again with elevated serum creatinine levels (4.26 mg/dL), proteinuria (protein-to-creatinine ratio (PCR) of 1,583 mg/g), peripheral edema, and hypertension. Two weeks before admission, the patient tested positive for severe acute respiratory syndrome coronavirus 2 (SARS-CoV-2) and was managed on an outpatient basis due to a mild disease course. Laboratory tests revealed pancytopenia (hemoglobin: 10.3 g/dL; platelets: 144 × 10^9^/L; leukocytes: 3.13 × 10^9^/L), low haptoglobin (<0.09 g/L), and elevated LDH levels (517 U/L). In a peripheral blood smear, schistocytes were observed. A re-biopsy of the transplant was performed and showed acute thrombotic microangiopathy without signs of rejection (Figures 1A,B). ADAMTS13 activity was normal, and tacrolimus trough levels were within the target range of 5–10 ng/mL prior to biopsy. An extended complement workup revealed alternative complement pathway dysregulation with reduced C3 and factor H levels, while classical and terminal pathway activation was unremarkable.

**Figure 2:**
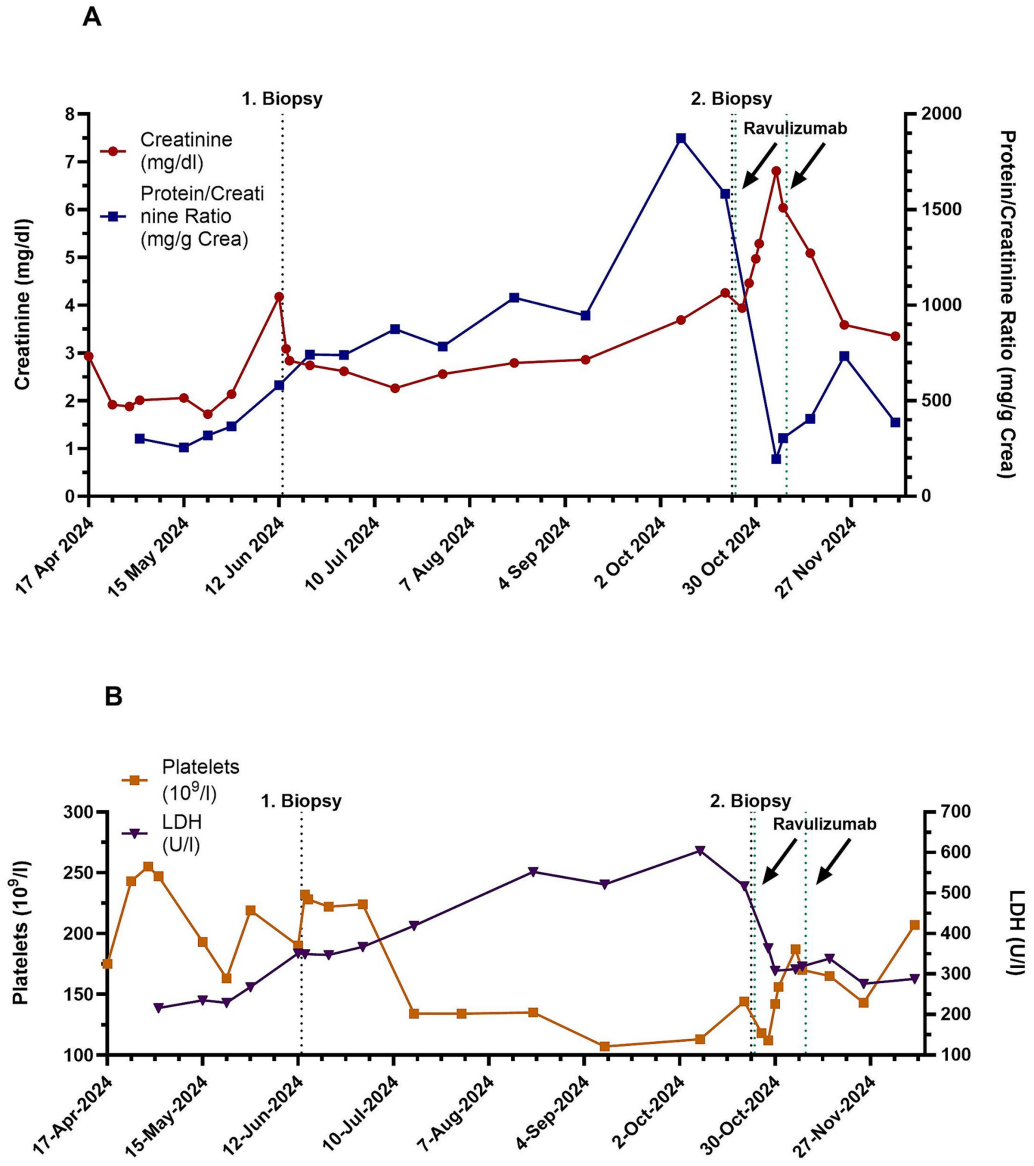
Serum creatinine and urinary protein-to-creatinine ratio (PCR) **(A)** and platelets and lactate dehydrogenase (LDH) activity **(B)** are shown over time.

The patient was administered with two doses of ravulizumab, each containing 2,700 mg, 2 weeks apart. Subsequently, kidney function improved, and markers of ongoing hemolysis were no longer detectable (Figures 2A,B). Genetic complement testing identified a heterozygous factor H mutation (Thr1217del), known to cause a quantitative deficiency in factor H protein and previously linked to CM-TMA (7–9). In retrospect, we suspect this mutation likely underpinned the kidney disease in both the patient and his sister, who was not available for genetic testing.

## Discussion

In this study, we report a case of COVID-19-triggered CM-TMA in the post-transplant setting that was successfully treated with ravulizumab.

TMA occurs in approximately 0.8–15% of kidney transplant recipients, most commonly within the first 3 years after transplantation (10), and may significantly affect graft and patient survival (11). This temporal pattern likely reflects the high frequency of potential triggers in the early post-transplant period, including ischemia–reperfusion injury, viral infections, antibody-mediated rejection, and exposure to immunosuppressive agents such as cyclosporin or tacrolimus (12, 13), which together may create a constellation of factors capable of precipitating CM-TMA. Similarly, COVID-19 may activate the complement cascade, particularly through the alternative pathway, and induce endothelial injury, thereby acting as a trigger for CM-TMA (14, 15).

This case provides several important clinical insights. First, although factor H is a key complement regulatory protein, pathogenic variants may remain clinically silent and undetected for decades. Consequently, genetic CM-TMA should remain in the differential diagnosis of post-transplant TMA regardless of the patient’s age.

Second, CM-TMA may follow an insidious course, making diagnosis difficult. The clinical spectrum of CM-TMA ranges from life-threatening systemic disease to less severe and renal-limited courses, which often require kidney biopsy for definitive diagnosis (1).

Third, our case raises the issue of end-stage kidney disease of unknown etiology or presumed “convenience diagnoses,” such as hypertensive nephropathy without histological confirmation. Such diagnoses may obscure an underlying, undetected disease with the potential to recur after transplantation (16).

Fourth, this case adds real-world evidence supporting the efficacy of ravulizumab in CM-TMA after kidney transplantation. Ravulizumab has been demonstrated to be both efficacious and safe in a wide variety of complement-mediated diseases, including CM-TMA (6). Compared with eculizumab, ravulizumab may improve quality of life because of its extended half-life and reduced infusion frequency (6).

Several limitations should be acknowledged. First, it is not possible to retrospectively establish the exact etiology of the patient’s original kidney disease. Second, genetic testing of the patient’s parents and sister was not feasible.

In summary, this case highlights a distinctive constellation of factors in CM-TMA, including apparent late manifestation despite genetic predisposition, although a previously unrecognized disease course leading to end-stage kidney disease cannot be excluded, COVID-19 as a potential triggering event, an insidious clinical course, and a rapid response to the long-acting C5 inhibitor ravulizumab.

## Data Availability

The original contributions presented in the study are included in the article/supplementary material, further inquiries can be directed to the corresponding author.
